# Prolonged Lifespan of Superhydrophobic Thin Films and Coatings Using Recycled Polyethylene

**DOI:** 10.3390/polym16131791

**Published:** 2024-06-25

**Authors:** Junaid Saleem, Zubair Khalid Baig Moghal, Gordon McKay

**Affiliations:** 1 Division of Sustainable Development, College of Science and Engineering, Hamad Bin Khalifa University, Qatar Foundation, Doha 5825, Qatar; 2 Center for Advanced Materials, Qatar University, Doha 2713, Qatar

**Keywords:** superhydrophobic, thin films, anti-aging, surface roughness, plastic waste

## Abstract

High-density polyethylene (HDPE) waste poses a significant environmental challenge due to its non-biodegradable nature and the vast quantities generated annually. However, conventional recycling methods are energy-intensive and often yield low-quality products. Herein, HDPE waste is upcycled into anti-aging, superhydrophobic thin films suitable for outdoor applications. A two-layer spin-casting method combined with heating-induced crosslinking is utilized to produce an exceptionally rough superhydrophobic surface, featuring a root mean square (RMS) roughness of 50 nm, an average crest height of 222 nm, an average trough depth of −264 nm, and a contact angle (CA) of 148°. To assess durability, weathering tests were conducted, revealing the films’ susceptibility to degradation under harsh conditions. The films’ resistance to environmental factors is improved by incorporating a UV absorber, maintaining their hydrophobic properties and mechanical strength. Our research demonstrates a sustainable method for upcycling waste into high-performance, weather-resistant, superhydrophobic films.

## 1. Introduction

Plastic waste recycling is a critical aspect of sustainable waste management, as the large amount of plastic waste generated annually is a major concern due to its non-biodegradable nature and potential harm to the environment [1,2,3,4]. Thermal and chemical recycling have emerged as promising methods to address the limitations of traditional mechanical recycling [5]. Thermal recycling of plastic waste encompasses several processes that convert plastics into useful products or energy by applying heat. The main types of thermal recycling include pyrolysis, gasification, incineration, and hydrothermal liquefaction [6]. Yet, thermal recycling has its drawbacks; the combustion process releases greenhouse gases and air pollutants. Additionally, incinerating plastic waste can produce toxic byproducts [7]. Chemical recycling, on the other hand, employs chemical reactions to depolymerize plastic waste into its monomers or other useful chemicals, allowing for the production of high-quality plastics identical to virgin materials [8]. Nonetheless, chemical recycling faces numerous challenges, such as elevated expenses and the necessity for a dedicated setup. Plastics recycled via chemical processes might not attain the same quality as virgin plastics, limiting their applicability for certain uses [9]. Recently, upcycling techniques have emerged as a strategy for converting plastic waste into value-added materials [10,11,12].

HDPE stands as one of the most extensively utilized polymers [13], with various studies emphasizing its potential for upcycling as a crucial strategy in mitigating environmental pollution. For instance, researchers have successfully synthesized an aerogel membrane by utilizing recycled polyethylene to address oil-water emulsions, employing methods such as swelling, solvent extraction, and freeze-drying [14]. Additionally, waste HDPE has been effectively transformed into oil sorbents using the extrusion-stretching technique, boasting both high selectivity and remarkable oil uptake capacity [15]. Moreover, initiatives have been undertaken to repurpose waste HDPE and PP in the creation of plastic bricks via compaction methods [16]. These examples demonstrate the feasibility and efficacy of upcycling HDPE waste into value-added products, contributing positively to environmental sustainability efforts.

Another application of utilizing waste HDPE is in the preparation of superhydrophobic films, especially those with prolonged aging and anti-weathering properties. In this context, a critical study has demonstrated the transformation of virgin HDPE into hierarchical nanostructured surfaces boasting superhydrophobic characteristics. Achieving a maximum static CA of 159° involves a sequential process comprising photolithography, aluminum etching, anodization, and polymer replication [16]. Also, thin film coatings using HDPE were applied to the silicon substrate to produce a superhydrophobic surface with a contact angle of 126° [17]. Moreover, a study investigated the properties of thin films of HDPE produced using the spin-coating technique to understand how the process parameters, such as the initial deposition temperature, affect the characteristics of the HDPE films, including their thickness, chemical composition, and morphology [18]. In addition, freestanding thin films and coatings utilizing HDPE [19], PP [20], and a combination of PP, HDPE, and low-density polyethylene (LDPE) waste [21] have been previously reported. These materials belong to the same class of polymers as HDPE, collectively known as polyolefins, further illustrating the broad applicability of waste plastic repurposing in the creation of superhydrophobic surfaces.

This study focuses on the upcycling of HDPE waste to create durable, superhydrophobic thin films suitable for outdoor use, addressing the issue of aging. We devised a two-layer fabrication approach using spin casting. Initially, a HDPE coating was applied to a glass substrate, exhibiting a CA of 148° but lacking mechanical strength. To enhance strength, heat treatment was applied, resulting in polymer crosslinking and a reduced CA of 102°. To maintain hydrophobicity while restoring strength, a second layer of HDPE was added without heat treatment, preserving surface roughness and bringing the CA back to 148°.

Durability assessments involved subjecting the films to weathering tests, which highlighted their susceptibility to degradation under harsh conditions. To counter this, we incorporated a UV absorber into the films, significantly improving their resistance to environmental factors. This ensured the retention of both hydrophobic properties and mechanical strength over time. Our findings demonstrate a sustainable approach to repurposing waste, resulting in the production of high-performance, weather-resistant superhydrophobic films.

## 2. Experimental

p-xylene from Sigma Aldrich (St. Louis, MA, USA) was utilized for the polymer dissolution. HDPE containers were collected from the local market. Chimassorb 81 was purchased from BASF, Florham Park, NJ, USA.

60 mg/mL of HDPE solution in Xylene was heated at 130 °C for 15 min or till a clear solution was obtained.

**Figure 1:**
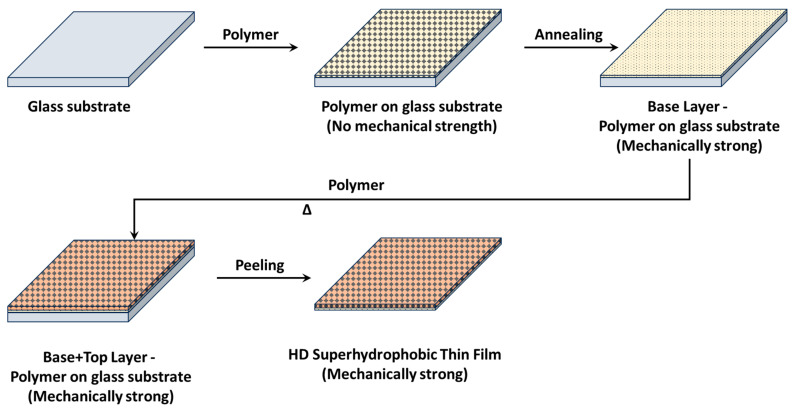
Showing the processes involved in the preparation of the superhydrophobic film.

The solution was then spin coated onto a substrate and heated at 130 °C on a heating plate for 5–15 s. After heating the base layer to enhance its strength, a second layer of HDPE was coated onto the base layer while it was hot. The top layer adhered well to the base layer because of the softening of the base layer, allowing for easy integration of the top layer. This approach achieves super-hydrophobicity in the film while preserving its mechanical strength, and it can be peeled off and applied anywhere. Figure 1 presents the processes involved in the preparation of the film.

Subsequently, to investigate the implementation of synthetic hydrophobic films in outdoor environments, a weather test was carried out. The films underwent weathering tests using a QUV/se accelerated weathering tester (Q-Lab, Westlake, OH, USA). This involved the use of fluorescent UV-A lamps that emitted light at a wavelength of 340 nm with an intensity of 0.76 W/m^2^. The samples underwent 8 h of UV irradiation at 50 °C, then 4 h of darkness at the same temperature with 100% condensing humidity. These cycles were repeated continuously on the thin film samples. The effects of the accelerated weathering were evaluated after 250 h of exposure.

The optical contact angle was determined using an OCA 35 from Dataphysics Instruments GmbH, located in Filderstadt, Germany. SEM images were obtained using an FEI Quanta650FEG (Thermo Fisher Scientific, Hillsboro, OR, USA). FTIR analysis was carried out with a PerkinElmer Frontier instrument. Thickness measurements were taken with a micrometer and verified with a Deflesko FS3 PosiTector 6000, which has a ferrous metal base. XRD studies were conducted with a PANalytical Empyrean multipurpose XRD instrument. XPS measurements were performed on a Thermo Fischer Escalab 250XI platform, utilizing a monochromated X-ray source (Al Kα: 1486.6 eV). AFM data was collected using an AFM from Oxford Instruments, based in Goleta, CA, USA.

## 3. Result and Discussion

### 3.1. CA

A CA greater than 90° signifies that the liquid droplet tends to exhibit minimal spreading on the solid surface. This behavior can be attributed to the dominant influence of cohesive forces within the liquid, resulting in a droplet shape that remains condensed and almost spherical. In this context, the intermolecular forces between the liquid and the solid, typically consisting of van der Waals interactions, become energetically unfavorable compared to the self-attractive forces within the liquid itself. The hydrophobic nature of the surface, as inferred from the larger CA, arises from the inadequate ability of the liquid to overcome the cohesive forces and establish a strong intermolecular interaction with the solid surface. Consequently, the liquid droplet tends to exhibit a distinct curvature, with the majority of its surface area resisting contact with the solid. The reduced interaction between the liquid and the solid surface imparts a repellent effect, rendering the surface resistant to wetting by water or other polar liquids.

**Figure 2:**
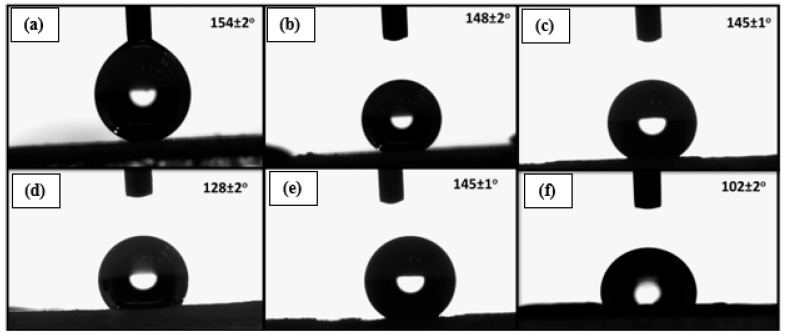
(**a**) CA of water in contact with the film; (**b**) CA of water dispensed on the surface with no UV absorber before weathering exposure; (**c**) CA of water dispensed on the surface with a UV absorber before weathering exposure; (**d**) CA of water dispensed on the surface with no UV absorber after weathering exposure; (**e**) CA of water dispensed on the surface with a UV absorber after weathering exposure; and (**f**) CA of water dispensed on the bottom heated surface.

Figure 2a illustrates the CA measurement of a hydrophobic film. Due to its high hydrophobicity, the water CA (WCA) is 154°. In Figure 2b, the CA of the film is shown before the application of a UV absorber. Subsequently, a weathering test was conducted to extend the film’s range of application for hydrophobicity. Two samples were taken for the weathering test, one coated with a UV absorber and the other left uncoated. The hydrophobic properties of the film were minimally affected by the application of the UV absorber, as the CA of the film with the UV absorber, prior to weathering exposure, was recorded as 145° (see Figure 2c), supporting its hydrophobicity. The second sample, which was not coated with a UV absorber, underwent the weathering test. After the weathering test, the CA was measured again (Figure 2d). It was observed that harsh weather conditions affected the hydrophobic properties of the films, resulting in a reduced CA of 128°. Later, the CA was measured for the sample that had been coated with a UV absorber prior to conducting the weathering test (see Figure 2e). It was noticed that the films retained their hydrophobicity when coated with a UV absorber, even under harsh weather conditions. The CA measurement of the UV-coated samples after the weathering test recorded 145°, which was almost the same as before the weather test. Figure 2f depicts the CA of the base layer of the film. The water CA for the base layer was very low, as it lost its hydrophobicity during the heating process. CAs of the thin films were also measured when they were heated at different temperatures, with a constant heating time of 25 min for all temperatures. Table 1 illustrates that as the heating temperature increased, the CA of the thin films gradually decreased, suggesting a reduction in their hydrophobicity, which is consistent with the previous study [22].

### 3.2. Surface Morphology

**Figure 3:**
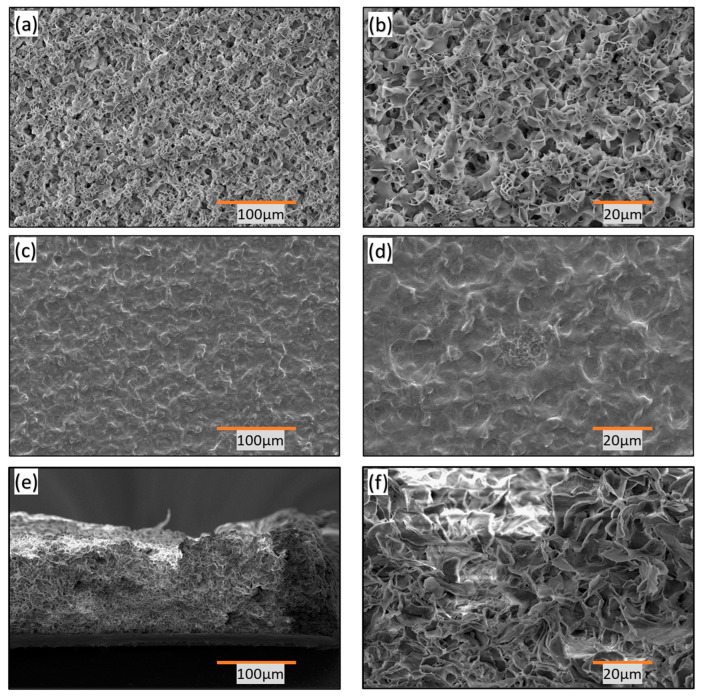
(**a**) Top view of the surface showing a homogenous surface with uniform pore size and pore-size distribution; (**b**) Top and close-views of the surface showing a uniform arrangement of floral petals; (**c**) Top view of the base layer showing closed pores and melted floral petals, resulting in a relatively smoother surface and a decreased CA of 102°; (**d**) Top and close-view of the surface showing the smooth surface caused by excess heating of the base layer to attain sufficient strength to be applied independently; (**e**) Cross sectional view showing the floral petal arrangement; and (**f**) Close view of cross section.

In Figure 3a. the porous nature of the top layer of the hydrophobic thin film is clearly visible, revealing a flaky structure on its surface. Figure 3b is an enlarged view of Figure 3a, which highlights the presence of spiky, sharp petal-like structures that contribute to the film’s superhydrophobic properties, as indicated by a CA of 148°.

The presence of petal-shaped pores in the top layer promotes hydrophobicity by facilitating the Cassie–Baxter phenomenon [23,24]. This phenomenon allows liquid droplets to rest on a textured or rough surface without fully wetting it. In the Cassie state, the droplet partially suspends itself on the surface, supported by air pockets trapped within the surface texture. This leads to the coexistence of solid and liquid phases at the interface. The Cassie state enhances the extreme water repellency of the film, making it useful in various applications such as anti-fouling coatings, microfluidics, waterproof fabrics, and biomedical devices.

To achieve hydrophobic properties and maintain its structural integrity, the film was engineered using a two-layer fabrication strategy. Figure 3d depicts the surface morphology of the base layer after heating. Heating the polymer softens it and results in a relatively smoother surface, characterized by closed pores and melted floral petals. The CA decreases to 102° in this case. Figure 3d also shows the top view and close-up of the hydrophobic surface, demonstrating the smooth surface achieved by subjecting the base layer to excessive heat treatment for increased strength and independent application.

Additionally, Figure 3e illustrates the cross-sectional view of the film, highlighting the arrangement of floral petals. Figure 3f provides a magnified view of the cross-section, revealing interconnected floral petal structures with a uniform distribution of pore size and spiky endings. These features contribute to the high CA observed and create air pockets that suspend water droplets, resulting in their repulsion from the surface.

### 3.3. Weathering Test

**Figure 4:**
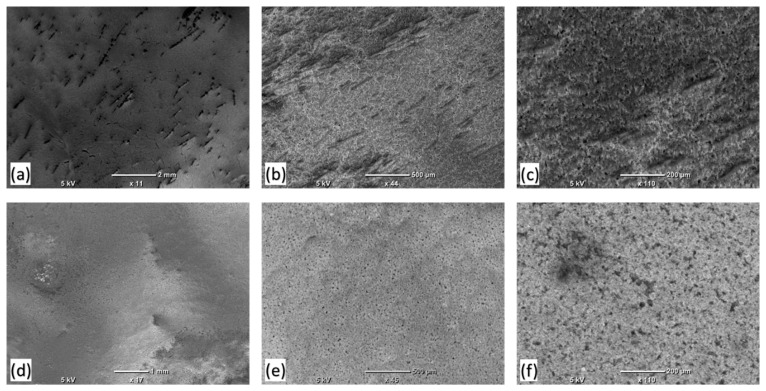
The SEM images of the HD films kept under weathering conditions to evaluate the morphological changes at the surface level. The figures (**a**–**c**) showed the HD top layer without UV absorber after the weathering test and (**d**–**f**) the top layer with UV absorber after the weathering test at different resolutions.

Maintaining excellent hydrophobicity even after exposure to harsh weather conditions is an additional quality exhibited by the films. Figure 4 illustrates the physical condition of the films, both with and without a UV absorber, when subjected to severe weather conditions. Initially, the films demonstrated the highest water CA of 148°. Subsequently, in order to broaden their outdoor applications, a UV absorber was added. Following the addition of the UV absorber, the water contact angle (WCA) of the films was measured to be 145°. Although the UV absorber slightly reduced the hydrophobicity efficiency of the films, it expanded their range of applicability. As depicted in Figure 4, the thin films easily deteriorated when exposed to harsh weather conditions in the weather chamber, as per the weathering test procedure. However, films coated with the UV absorber did not deteriorate and maintained their hydrophobic properties. Furthermore, wettability analysis confirmed that the CA of the UV absorber-coated thin films was retained even after exposure to harsh weathering conditions.

### 3.4. Surface Roughness

The hydrophobic property, also known as anti-wetting, is determined by the CA exhibited by a surface. When the CA (θ) of a surface is equal to or greater than 90°, it demonstrates anti-wetting characteristics, whereas a hydrophilic surface has a CA of less than 90°. The CA primarily relies on surface topography, including roughness and cleanliness. Increasing the roughness of an initially hydrophobic surface is one method to enhance its hydrophobic properties.

**Figure 5:**
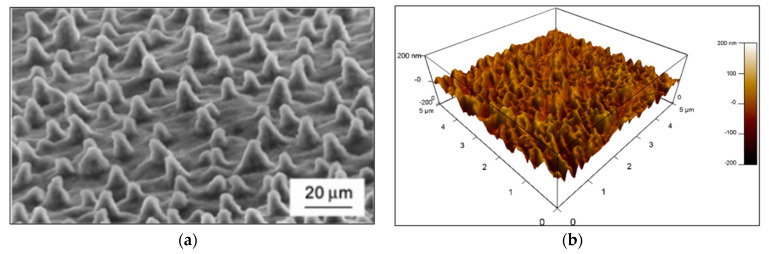
(**a**) SEM image of a leaf. (**b**) AFM image of the film.

A rough surface provides a larger solid–liquid interface area, leading to higher overall energy. This effect causes an increase in the CA for hydrophobic surfaces and a decrease in the CA for hydrophilic surfaces. In designing hydrophobic surfaces, materials scientists frequently take inspiration from naturally occurring highly hydrophobic surfaces, like those of the lotus leaf. Figure 5a shows an image of the lotus leaf, highlighting its unique large bumps, or papillae [25]. Interestingly, this image bears a resemblance to our AFM image (Figure 5b).

**Figure 6:**
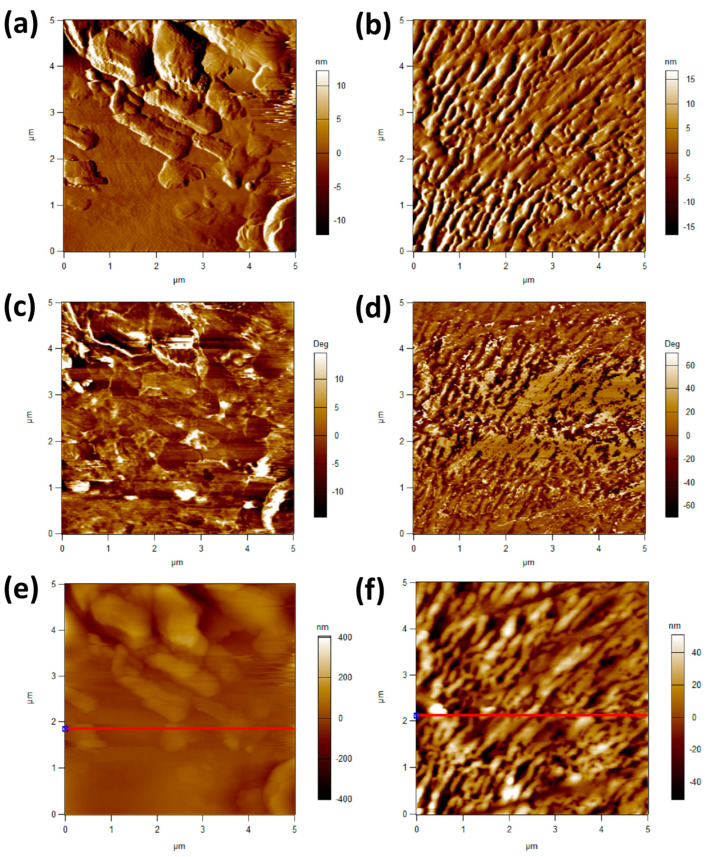
(**a**,**b**) represent amplitude retrace mode, (**c**,**d**) represent phase retrace mode, and (**e**,**f**) represent the Z-sensor retrace method. (**a**,**c**,**e**) represent the smooth surface or the base layer, and (**b**,**d**,**f**) represent the rough superhydrophobic surface. AFM of the top layer showing rougher surface and base layer showing relatively smoother surface.

The roughness of their surfaces can significantly influence the hydrophobic properties of thin films [26]. When a surface is rough, it has the potential to create air pockets between itself and a water droplet; this phenomenon is known as the Cassie–Baxter state [23,24]. The presence of these air pockets is clearly evident in Figure 6, which displays an AFM image.

**Figure 7:**
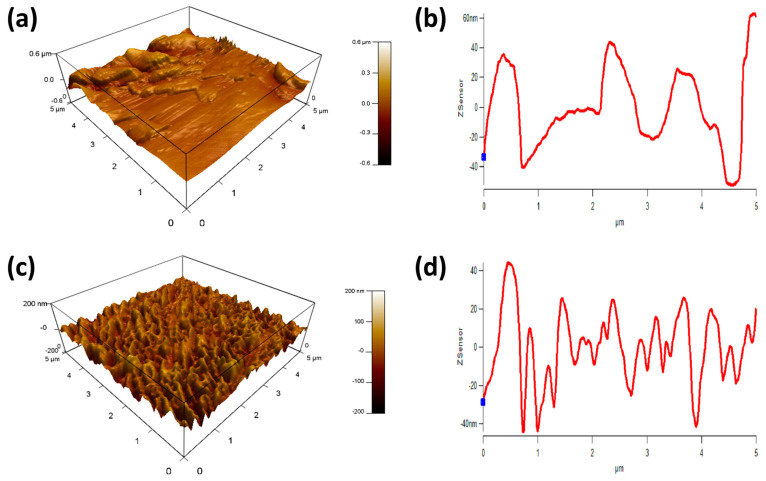
(**a**,**b**) represent the smooth surface or the base layer, and (**c**,**d**) represent the rough superhydrophobic surface; (**a**,**c**) represent 3D- XYZ axes imaging, and (**b**,**d**) represent 2D XZ axes imaging.

On the other hand, if the surface is smooth, water droplets can spread and saturate it more easily, which diminishes the surface’s hydrophobicity. Consequently, the hydrophobicity of the base layer decreased when it was heated. This can be observed through the comparison of the AFM images of the top and base layers, where the image of the base layer appears smoother, as shown in Figure 7. The RMS value for the top layer was 50 nm, while the base layer exhibited a lower RMS value of only 20 nm, as indicated in Table 2.

### 3.5. XPS

**Figure 8:**
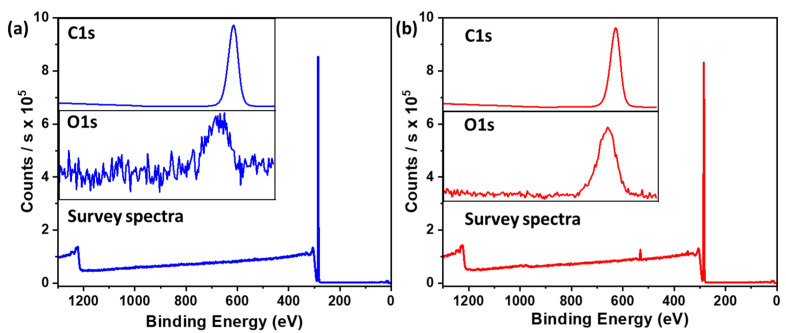
XPS spectra of the HD superhydrophobic thin film (**a**) top layer and (**b**) base layer.

The films’ composition was analyzed using XPS, with the results displayed in Figure 8. This figure indicates the presence of oxygen in the base layer of the film. In contrast, the spectra for the top layer show no presence of oxygen in the films. Initially, when the hydrophobic film was prepared, it exhibited insufficient strength. To enhance the strength of these thin films, they were subjected to heat treatment, resulting in oxidation. As a consequence of heating, the thin films acquired a free-standing nature. Subsequently, the top layer was applied without undergoing any heating treatment. The spectra of the top layer did not exhibit any oxygen peaks.

### 3.6. FTIR

**Figure 9:**
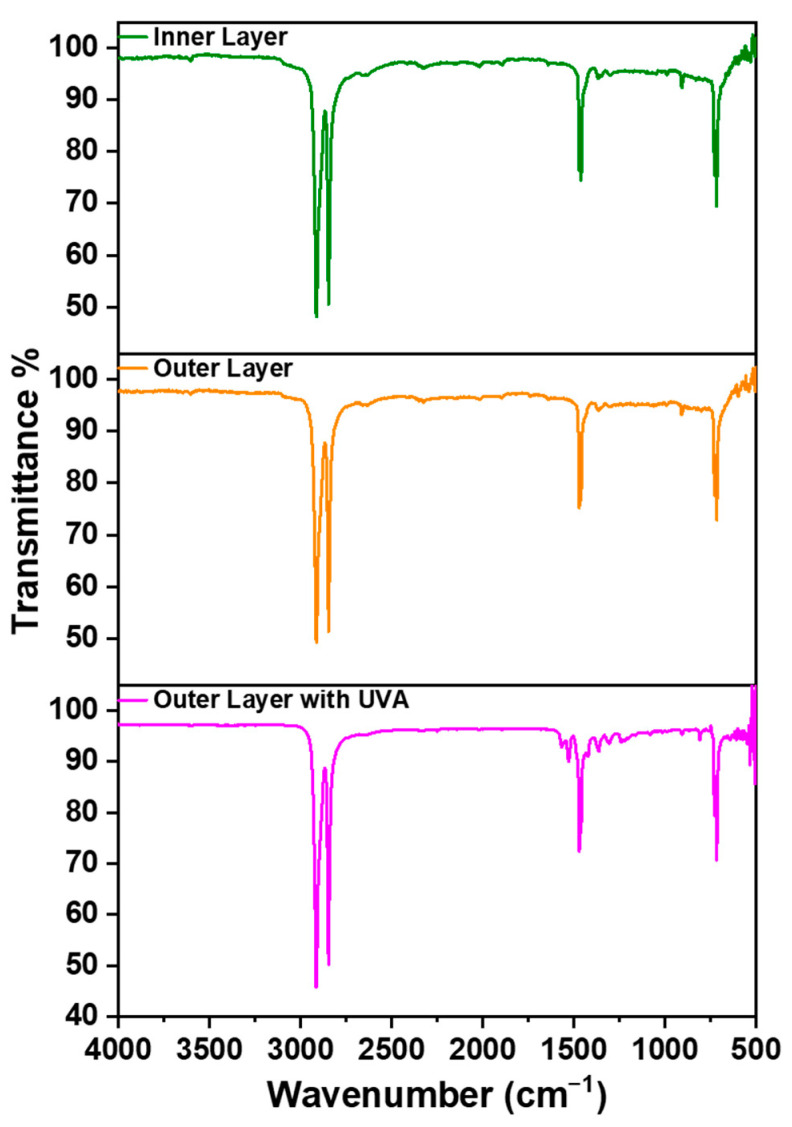
FTIR analysis of the inner (base) and outer (top) layers with and without UV absorbers.

The FTIR results are presented in Figure 9. The spectral peaks confirmed the presence of the C-H group in both the top layer and the base layer, while the top layer with UV-absorber exhibited peaks within the range of 3000 cm^−1^ to 2850 cm^−1^. Additionally, there were peaks observed at 1420 cm^−1^, indicating the presence of the O-H group in all layers. Furthermore, the peaks identified at 750 cm^−1^ confirmed the existence of C=C bonds. Notably, in the FTIR spectrum of the top layer with UV absorber, there were additional small peaks in the vicinity of 750 cm^−1.^ These peaks were associated with the internal chemistry of the UV absorber.

The stretching of C-C bonds at 750 cm^−1^ can be attributed to changes in crystallinity during degradation induced by heating. The heating process led to the degradation of the films, resulting in the cleavage of polymer chains and structural deterioration. During the degradation of the polymer, the generation of free radicals occurred, which then reacted with oxygen in the surrounding environment, yielding peroxide groups [27,28].This phenomenon likely accounts for the observed continuous increase in the polar component and the corresponding increase in CA on the heated surface of the base layer. Furthermore, a gradual decrease in intensity was observed in the base layer spectra as it was heated, ultimately leading to a decrease in crystallinity and a transition to the amorphous phase of the films.

### 3.7. XRD

**Figure 10:**
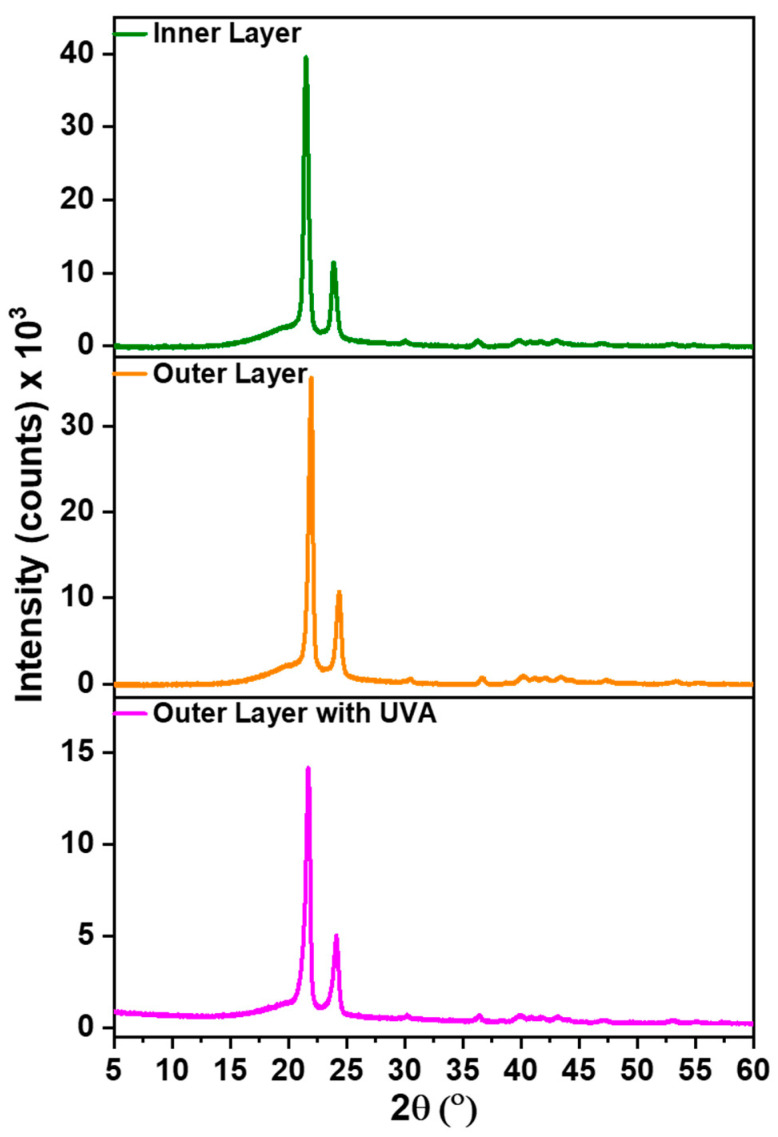
XRD spectra of the outer (top) layer, the inner (base) layer, and the UV-Absorber coated outer (top) layer.

Figure 10 presents the XRD results for the top layer, base layer, and top layer with a UV absorber. The XRD spectra of all three samples exhibited two prominent characteristic peaks of HDPE at 21° and 25°. The presence of sharp peaks suggests a high degree of crystallinity.

Crystallinity significantly influences the surface roughness of HDPE. As a semi-crystalline polymer, it consists of both crystalline and amorphous regions. The crystalline regions are characterized by an organized and tightly packed molecular arrangement, whereas the amorphous regions lack long-range order. When the base layer is heated, the molecular arrangement within the polymeric structure becomes disrupted. Upon cooling from a molten state to a solid state, the polymer chains reassemble into crystal structures [29]. These crystals confer rigidity and strength to the material. The crystalline structure of the polymer influences its mechanical properties, including surface roughness. The presence of crystalline regions leads to irregularities on the surface, resulting in a rough texture, which contributes to the material’s hydrophobicity.

### 3.8. DSC

**Figure 11:**
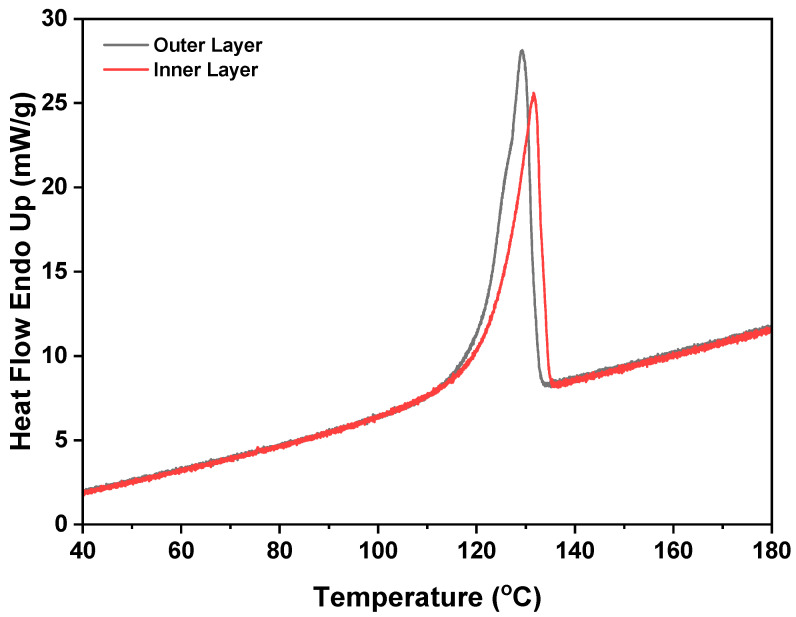
Thermal profile of the outer (top) and inner (base) layers.

The thermal analysis presented in Figure 11 compares the thermal behavior of the outer (top) and inner (base) layers. While the inner (base) layer undergoes annealing, the outer (top) layer does not. As a result of annealing, the films exhibit a higher degree of crystallization, as evidenced by their elevated melting temperature. During annealing, the polymer chains within the films undergo rearrangement and reorientation, leading to significant changes in surface morphology and chemical structure.

### 3.9. Tensile Strength and Modulus

**Figure 12:**
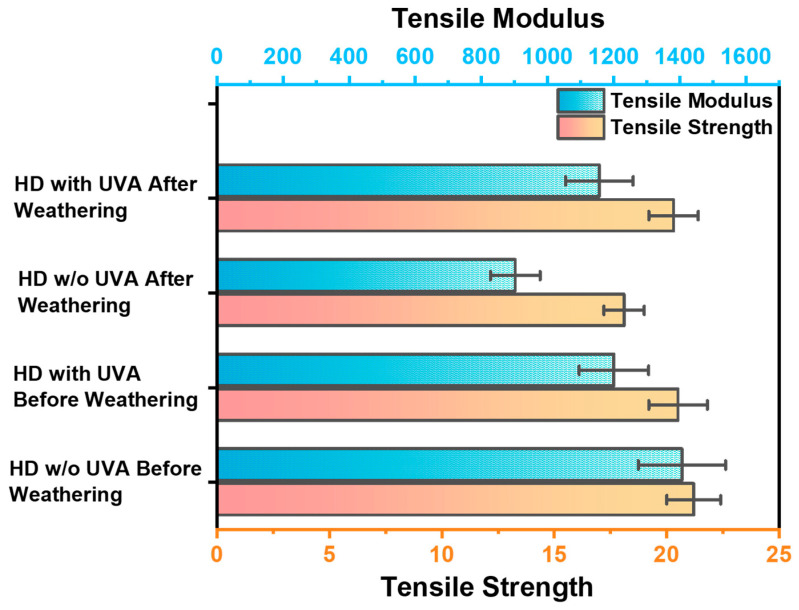
Tensile strength and modulus of the films with and without UV-Absorbers before and after weathering exposure.

Figure 12 illustrates the tensile strength and modulus of the films. Initially, before subjecting the samples to a weather test and without the application of any UV absorber, the tensile strength was recorded at 21 MPa, with a tensile modulus of 1406 MPa. However, after exposing the same samples to the weather test without a UV absorber, both the tensile strength and modulus decreased to 18 MPa and 902 MPa, respectively. To bolster the films’ strength in harsh environments, a UV absorber was introduced. After applying the UV absorber, the tensile strength and Young’s modulus were measured at 20 MPa and 1200 MPa, respectively. Following the weathering test on the UV absorber-coated samples, the tensile strength remained at 20 MPa, and the modulus was recorded at 1156 MPa. These results demonstrate the effectiveness of the UV absorber in enhancing the performance of the films under weathering conditions, with minimal impact on their strength and hydrophobicity, and are in line with environmental management practices [30].

## 4. Conclusions

This study demonstrates the upcycling of HDPE waste into durable superhydrophobic thin films using a two-layer spin-casting method combined with heating-induced crosslinking. The dual-layer fabrication approach maintained a high surface roughness essential for superhydrophobicity. The base layer, subjected to heating, provided the necessary strength, while the unheated top layer preserved the required surface texture to achieve a high CA of 148°.

The durability of these films under outdoor conditions has been a critical focus of this work. Incorporating a UV absorber significantly enhanced the weather resistance of the films. While uncoated films showed deterioration and reduced hydrophobicity after weathering tests, those coated with the UV absorber retained their hydrophobic properties and mechanical strength, demonstrating a CA of 145° post-weathering.

The UV absorber not only protected the films from environmental degradation but also preserved their tensile strength and Young’s modulus, ensuring the films’ longevity and effectiveness in harsh outdoor conditions. This resilience is a testament to the films’ suitability for long-term outdoor applications, where they can provide reliable performance and protection against water-based contaminants.

This study presents a viable and sustainable method for converting HDPE waste into high-quality, superhydrophobic thin films. These films, with improved durability and resistance to weathering, hold great potential for various outdoor applications, contributing to environmental sustainability and the advancement of polymer recycling technologies.

## Figures and Tables

**Table 1:** WCA measurement at different temperatures.

Temperature (°C)	Heating Time (min)	Water CA Top Layer
25	25	148° ± 2°
45	25	145° ± 1.3°
65	25	141° ± 1.2°
85	25	138° ± 1.3°
105	25	114° ± 2.5°
130	25	103° ± 0.8°

**Table 2:** RMS parameter for AFM analysis of the films.

Parameters	Base Layer (nm)	Top Layer (nm)
RMS	20	50
Max	86	222
Min	−74	−264
Avg Deviation	16	35

## Data Availability

The original contributions presented in the study are included in the article, further inquiries can be directed to the corresponding author/s.
